# Supplementary material for: Association between oral microbiome and seven types of cancers in East Asian population: a two-sample Mendelian randomization analysis
Source: Front Mol Biosci. 2023 Nov 21;10:1327893. doi: 10.3389/fmolb.2023.1327893 (PMC10702768; doi:10.3389/fmolb.2023.1327893)
Supplement: Supplementary file 1 [file DataSheet1.ZIP › Supplementary Materials/MR plots for tongue/tongue═╝/Lung cancer/pheno.2550_to_lung cancer_leave_one_out.pdf]

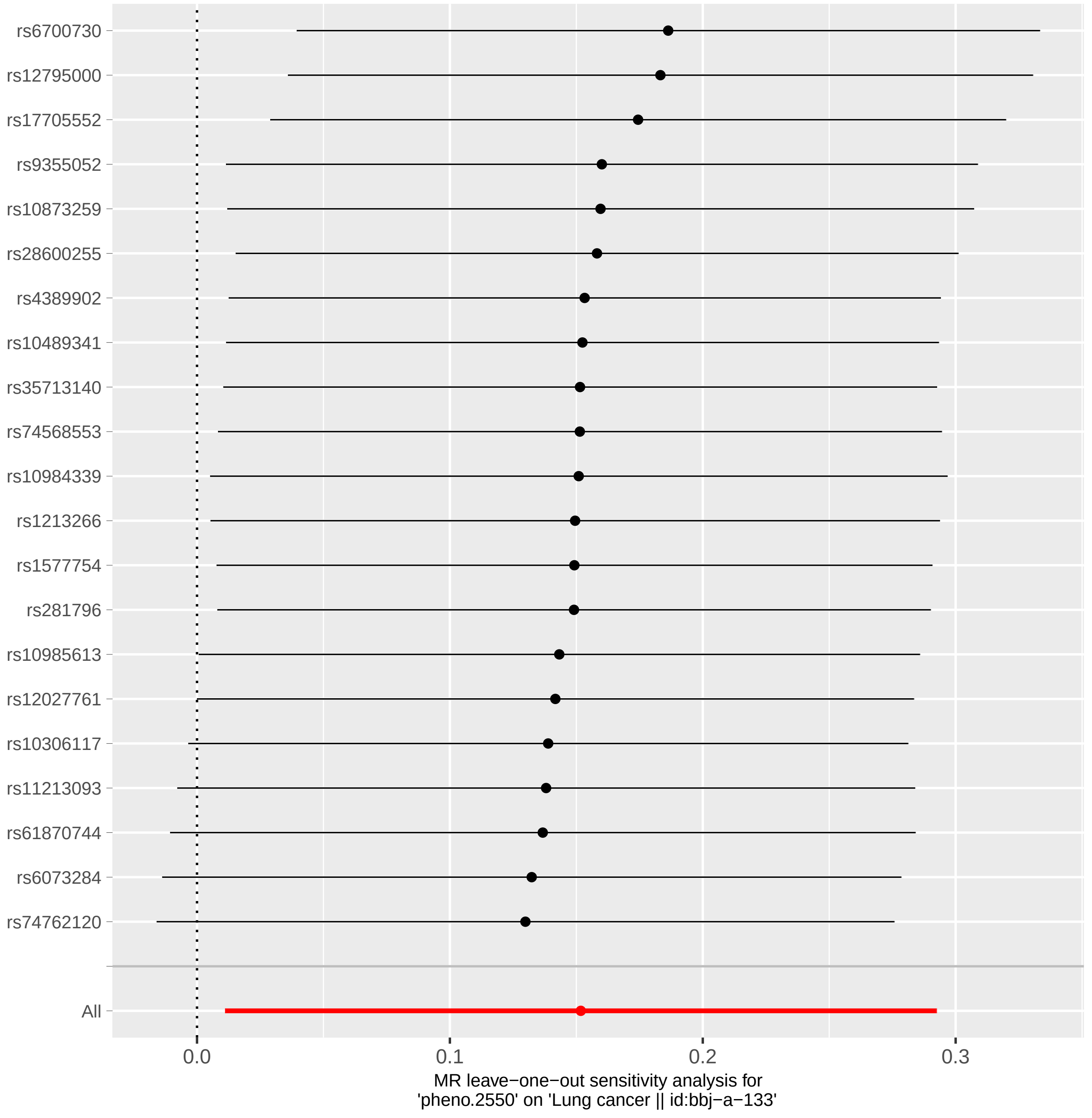

rs6700730
rs12795000
rs17705552
rs9355052
rs10873259
rs28600255
rs4389902
rs10489341
rs35713140
rs74568553
rs10984339
rs1213266
rs1577754
rs281796
rs10985613
rs12027761
rs10306117
rs11213093
rs61870744
rs6073284
rs74762120
All
0.0
0.1
0.2
0.3
MR leave−one−out sensitivity analysis for
'pheno.2550' on 'Lung cancer || id:bbj−a−133'
